# Immune Response to Therapeutic Staphylococcal Bacteriophages in Mammals: Kinetics of Induction, Immunogenic Structural Proteins, Natural and Induced Antibodies

**DOI:** 10.3389/fimmu.2021.639570

**Published:** 2021-06-14

**Authors:** Zuzanna Kaźmierczak, Joanna Majewska, Paulina Miernikiewicz, Ryszard Międzybrodzki, Sylwia Nowak, Marek Harhala, Dorota Lecion, Weronika Kęska, Barbara Owczarek, Jarosław Ciekot, Marek Drab, Paweł Kędzierski, Marta Mazurkiewicz-Kania, Andrzej Górski, Krystyna Dąbrowska

**Affiliations:** ^1^ Research and Development Center, Regional Specialist Hospital, Wroclaw, Poland; ^2^ Bacteriophage Laboratory, Hirszfeld Institute of Immunology and Experimental Therapy, Polish Academy of Sciences, Wroclaw, Poland; ^3^ Department of Clinical Immunology, Transplantation Institute, Medical University of Warsaw, Warsaw, Poland; ^4^ Laboratory of Microscopic Techniques, University of Wroclaw, Wroclaw, Poland; ^5^ Department of Experimental Oncology, Hirszfeld Institute of Immunology and Experimental Therapy, Polish Academy of Sciences, Wroclaw, Poland; ^6^ Unit of Nano-Structural Bio-Interactions, Hirszfeld Institute of Immunology and Experimental Therapy, Polish Academy of Sciences, Wroclaw, Poland; ^7^ Advanced Materials Engineering and Modelling Group, Faculty of Chemistry Wroclaw University of Science and Technology, Wroclaw, Poland; ^8^ Department of Animal Developmental Biology, University of Wroclaw, Wroclaw, Poland

**Keywords:** bacteriophage, *Staphylococcus aureus*, antibodies, immune response, phage therapy

## Abstract

Bacteriophages are able to affect the human immune system. Phage-specific antibodies are considered as major factors shaping phage pharmacokinetics and bioavailability. So far, general knowledge of phage antigenicity nevertheless remains extremely limited. Here we present comparative studies of immunogenicity in two therapeutic bacteriophages, A3R and 676Z, active against *Staphylococcus aureus*, routinely applied in patients at the Phage Therapy Unit, Poland. Comparison of the overall ability of whole phages to induce specific antibodies in a murine model revealed typical kinetics of IgM and IgG induction by these two phages. In further studies we identified the location of four phage proteins in the virions, with the focus on the external capsid head (Mcp) or tail sheath (TmpH) or an unidentified precise location (ORF059 and ORF096), and we confirmed their role as structural proteins of these viruses. Next, we compared the immune response elicited by these proteins after phage administration in mice. Similar to that in T4 phage, Mcp was the major element of the capsid that induced specific antibodies. Studies of protein-specific sera revealed that antibodies specific to ORF096 were able to neutralize antibacterial activity of the phages. In humans (population level), none of the studied proteins plays a particular role in the induction of specific antibodies; thus none potentially affects in a particular way the effectiveness of A3R and 676Z. Also in patients subjected to phage therapy, we did not observe increased specific immune responses to the investigated proteins.

## Introduction

Staphylococcal infections are among the most challenging health disorders, particularly threatening due to the frequent antibiotic resistance, known as MRSA (Methicillin-resistant *Staphylococcus aureus*). Anti-staphylococcal phages capable of combating infections caused by *Staphylococcus* spp. promise a particularly useful alternative to antibiotics due to their high efficacy against this pathogen (1–3). Importantly, their targeting efficacy includes drug-resistant staphylococcal infections caused by MRSA strains, as we demonstrated before (4). Nearly 600 anti-staphylococcal phages have been described (NCBI, 2020); all of them belong to tailed phages, Caudovirales, with double-stranded DNA and a distinct icosahedral head (5). Independent of subtype, anti-staphylococcal phages demonstrate good antibacterial activity (6, 7). As we reported previously at the Phage Therapy Unit of the Hirszfeld Institute of Immunology and Experimental Therapy, Polish Academy of Science (PTU HIIET PAS), as much as 36.7% of otherwise incurable infections by staphylococci have been successfully treated with phages (4). Importantly, clinical observations and laboratory data unanimously confirm the general safety of phage therapy (4, 6, 7).

Phages are capable of affecting the human immune system, both as therapeutic agents and as part of the natural mammalian microbiome (8). Many studies of the immune response to phages have been conducted in animal models where specific antibodies are typically observed after phage administration (9–15). In humans, the presence of phage-specific antibodies was observed as resulting from natural contact with phages (14, 16), or as phage-specific antibody production after therapeutic administration of phage, as could be concluded from long-term practice of HIIET PAS (14, 16–18). Interestingly and importantly, induction of anti-phage antibodies in the course of phage therapy does not exclude a favorable effect of phage treatment (17).

Though phage ability to induce specific antibodies has been observed many times, data on how particular structural proteins in virions contribute to anti-phage antibody induction are very scarce. They relate mainly to T4, which is a model phage, not recommended for therapeutic use, mainly due to the relatively high frequency of phage-neutralizing antibodies in the human population (14, 15, 19). The anti-staphylococcal phages, important tools in combating staphylococcal infections (including MRSA), have never been studied in humans in terms of the immunogenicity of their individual proteins. Therefore, we addressed this key issue in our present study.

Here we present a detailed analysis of antibody induction by two phages, A3R and 676Z, which are therapeutic agents against *Staphylococcus aureus* and which represent closely related strains (4, 20). Comparison of A3R genome vs 676Z revealed the high similarity over 99% of these genomes, except additional 7.5 kbp long fragment in 676Z sequence. The biological role of this additional fragment has not been identified yet, however these phages do not demonstrate identical specificity to bacterial hosts (17, 18). We studied the specific immune response induced by these phages and by their structural proteins, in a healthy human population and in patients subjected to phage therapy at the PTU. We assessed the frequency of naturally-occurring phage-neutralizing antibodies in a healthy human population and we dissected the roles of individual proteins as structural units of the phage virion in profiling protein-specific IgG levels in a human population. We assumed that since *Herelleviridae* constitute a significant component of the natural human phageome, the human population is not naïve towards *Herelleviridae* phage proteins. We predicted cross-reactivity in humans for exposed capsid elements. For this purpose, in addition to known key capsid proteins, we searched for so far unknown candidates. We report for the first time the precise location of two proteins of hitherto unknown position in the capsid and demonstrated their locations exposed on the virion and natural immunity in a healthy human population. In addition to humans, we included mice in this analysis because high-dose antigenic stimulation and specific protein stimulation could not be achieved in humans. In mice, a set of specific sera applicable for immunogold electron microscopy (EM) localization were developed and used to determine the localization of major capsid and tail proteins, as well as two proteins of hitherto unknown localization. Overall levels of whole phage-specific and protein-specific antibodies were also assessed in the group of patients subjected to phage therapy.

## Materials and Methods

### Ethics Approval Statement

All animal experiments were performed according to EU Directive 2010/63/EU for animal experimentations and were approved by the 1^st^ Local Committee for Experiments with the Use of Laboratory Animals, Wroclaw, Poland (No. 56/2017). The authors followed the ARRIVE (Animal Research: Reporting of *in vivo* Experiments) guidelines (21).

Plasma of healthy donors and PTU patients undergoing phage therapy were collected according to the Bioethics Committee at the Wroclaw Medical University, Poland (approval No. 530/2011 and 797/2017).

### Staphylococcal Bacteriophages A3R and 676Z

Phages A3R (GenBank, accession number JX080301.2) and 676Z (GenBank, accession number JX080302.2) were obtained from the collection of Hirszfeld Institute of Immunology and Experimental Therapy, Polish Academy of Science (HIIET PAS). The bacterial host for A3R is *S. aureus* strain R19930, for 676Z *S. aureus* strain Z11778, obtained from the Polish Collection of Microorganisms (HIIET PAS, Poland). Phages are taxonomically classified as *Herelleviridae, Twortvirinae*, *Kayvirus*; Staphylococcus virus G1. The level of A3R and 676Z identity of genomes is 94.6% (20).

To prepare phage lysates, flasks containing enriched nutrient broth were inoculated with bacterial host suspension and incubated at 37°C for 3 h with shaking. Then, phages were added to the flasks, the cultures were kept for 30 min at room temperature (RT), to allow for phage adsorption to the host and then incubated at 37°C with shaking for 10 h. After that time the flasks were placed at 4°C and left for 48 hours to clarify. Phage lysates were then centrifuged at 8000 rpm, the supernatants were filtered through 0.22-μm Millipore membrane filters (Merck Millipore) and purified using Fast Protein Liquid Chromatography (FPLC) on Sepharose 4B (Sigma-Aldrich), detailed information on timing and absorbance was presented in **Supplementary Figure S2** in Supplementary section (**Supplementary Figure S2**). The endotoxin removal from phage preparations was done with EndoTrap HD (Hyglos GmbH, Germany). Such preparations were subjected to dialysis through 1,000 kDa membranes (Spectrum Laboratories, USA) against phosphate-buffered saline (PBS) (Na2HPO4 6.5 uM, KCl 3 mM, KH2PO4 1.5 mM, NaCl 137 mM, Na2HPO4x12H2O 8.1 uM) (14, 15, 22, 23). Phage titers were determined using serial dilutions and the double-layer agar plates technique according to Adams (24). Such preparations were used for animal experiments, and further tests (ELISA, blocking).

### Phage Proteins

Phage proteins were used to obtain protein-specific sera and as bottom antigens in the ELISA test and in microarray experiment. Five proteins derived from staphylococcal phages were used: Mcp – major capsid protein (product of gene AFN38122.1 or AFN38316.1 for A3R and 676Z, respectively), TmpH – tail morphogenetic protein H (product of gene AFN38181.1 or AFN38375.1 for A3R and 676Z, respectively), gpORF059 (product of gene AFN113.1 or AFN307.1 for A3R and 676Z, respectively), ORF096 (product of gene AFN38152.1 or AFN38346.1 for A3R and 676Z, respectively), ORF123 (product of gene AFN176.1 or AFN370.1 for A3R and 676Z, respectively) and two proteins derived from T4 phage: gp23 and Hoc (15). Proteins were expressed from expression vectors pDEST15 (Mcp) or pDEST24 (TmpH, ORF096, ORF059, ORF123) (Thermo Fisher) in *E. coli* B834(DE3) F− ompT hsdSB(rB− mB−) gal dcm met (DE3) (Novagen) grown in Luria-Bertani Broth (LB) high salt (10 g/L of NaCl) culture medium (Sigma-Aldrich) with ampicillin, chloramphenicol and 3 mM L-arabinose (Sigma-Aldrich) at 37°C until OD600 reached 0.8. For proper folding of proteins, chaperones groES and groEL (from pGRO7 vector, TaKaRa Bio Inc.) were used. Expression of phage proteins was induced with 0.2 mM isopropylthio-b-D-galactoside (IPTG) (Thermo Scientific) and conducted overnight at 25°C. Cultures were then centrifuged for 5 min at 8000 rpm. The supernatant was removed. Harvested bacteria were suspended in phosphate buffer (50 mM Na_2_HPO_4_, 300 mM NaCl, pH 8.0), treated with phenylmethylsulfonyl fluoride (1 mM) and incubated on ice for 15 min. The lysis was performed by incubation with lysozyme (0.5 mg/ml) for 6–7 h on ice and by the freeze-thaw method (80°C). The preparation was then supplemented with Mg^2+^ (up to 0.25 mM), DNase (10 mg/ml) and RNase (20 mg/ml), and incubated on ice for 3 h. Fractions were separated by double centrifugations (12 000 rpm, 45 min, 15°C). The soluble fraction was filtered through 0.45 µm polyvinylidene difluoride (PVDF) filters and incubated with glutathione sorbent slurry (Glutathione Sepharose 4B, GE Healthcare Life Sciences), washed with phosphate buffer, and proteins were released by proteolysis with AcTev protease (5 U/mL) (Pure Biologics, Poland) at 10°C; GST tags remained bound in the resin. The first step of lipopolysaccharide (LPS) removal from all protein preparations was done with EndoTrap HD (Hyglos GmbH, Germany). Gel filtration FPLC (fast protein liquid chromatography) was performed on a Superdex 75 10/300 GL column (GE Healthcare Life Sciences, Poland). The final step of LPS removal was performed with EndoTrap blue (Hyglos GmbH). The sample was dialyzed against phosphate-buffered saline (PBS) and filtered through 0.22-μm PVDF filters (Millipore). Proteins were assessed by SDS-PAGE and concentrations were determined by the Lowry chromogenic method (Thermo Scientific, USA). Native protein conformation was positively verified by reaction with reference sera specific to whole phage virions.

### Specific Anti-Phage Antibody Level Measurement by ELISA

MaxiSorp flat-bottom 96-well plates (Nunc, Thermo Scientific) were coated with highly purified phage preparations at 5×10^8^ pfu per well, overnight at 4°C. Subsequently, wells were washed 5 times with PBS and blocked with 5-fold diluted SuperBlock Blocking Buffer (Thermo Scientific) in PBS at RT for 45 minutes. Blocking solution was then removed and plates were washed 5 times with PBS with 0.05% Tween 20 (BD Biosciences). Plasma was diluted in PBS, then added to wells at 100 µl per well and incubated at 37°C for 2 h. Each sample was investigated in duplicate. Subsequently, plates were washed 5 times with PBS with 0.05% Tween 20 and 100 µl per well of diluted detection antibody: horseradish peroxidase-conjugated goat anti-mouse IgM (Jackson ImmunoResearch Laboratories) or IgG (Jackson ImmunoResearch Laboratories) or anti-human IgG (Abcam, Ab81202) was applied to the plates and incubated for 1h at RT in the dark. The antibody solution was removed and the plates were washed 5 times with PBS with 0.05% Tween 20. 3,3′,5,5′-Tetramethylbenzidine (50 μl/well) was used as a substrate reagent for peroxidase according to the manufacturer’s instructions (R&D Systems) and incubated for 30 min. Finally, 25 µl of 2N H_2_SO_4_ was added to stop the reaction and the absorbance was measured at 450 nm (main reading) and normalized by subtracting the background absorbance at 570 nm.

### Measurement of Protein Content in 96-Well Plates

The CBQCA Protein Quantitation Kit (Thermo Fisher) was adapted to measure protein content in wells of MaxiSorp flat-bottom 96-well plates and determine optimal phage protein concentrations for plate coating. Due to each protein’s individual ability to adhere to the surface of the plastic plate, conditions for adsorption of each protein were optimized before ELISA procedure by CBQCA. Plates were coated with phage proteins at various concentrations overnight at 4°C. PBS was added to wells serving as the blank (6 wells) and standard curve. Plates were washed 5 times with PBS. Eighty µl of assay buffer (0.1 M sodium borate buffer, 0.1% Triton X-100, pH 9.3) supplemented with 0.83 mM KCl were added to protein-coated and blank wells. A six-point standard curve of 160 ng, 80 ng, 40 ng, 20 ng, 10 ng and 5 ng per well of bovine serum albumin (BSA) in the assay buffer supplemented with 0.83 mM KCl was prepared in designated wells. Subsequently, 20 µl of 1 mM ATTO-TAG CBQCA reagent solution in the assay buffer were added to each well. The plate was covered from light and incubated for 2h at RT with shaking (400 rpm). Fluorescence was read using an excitation/emission wavelength of approximately 465/550 nm. The mean blank value was calculated and subtracted from the reads. Protein content in wells was calculated with reference to the standard curve obtained for BSA: Mcp 0.8 µg/well, TmpH 1.5 µg/well, gpORF059 1.5 µg/well, gpORF096 0.2 µg/well, and gpORF123 0.4 µg/well. These amounts allowed for similar amounts of proteins that covered well surface: 23 (+/-5) ng of each investigated protein.

### Immunogenicity of Individual Protein Assessment by ELISA

MaxiSorp flat-bottom 96-well plates (Nunc, Thermo Scientific) were coated with highly purified protein preparations (Mcp: 0.8 µg/well, TmpH: 1.5 µg/well, gpORF059: 1.5 µg/well, gpORF096: 0.2 µg/well, gpORF123: 0.4 µg/well) overnight at 4°C. Subsequently, wells were washed 5 times with PBS and blocked with 5-fold diluted SuperBlock Blocking Buffer (Thermo Scientific) at RT for 45 minutes. Blocking solution was then removed and plates were washed 5 times with PBS with 0.05% Tween 20. Plasma samples diluted in PBS were then added to wells at 100 µl per well. Two standard curves were prepared on each plate: one with Mcp-, TmpH-, ORF059-, ORF096 or ORF123-coated wells and their respective reference sera. Each curve consisted of 10 points of two-fold reference plasma dilutions, from 1:100 to 1:51,200 (each dilution was processed in duplicate) and two uncoated wells to which PBS was added instead of plasma samples that served as blank. Further steps of the assay were performed as described above. Gen5 software was used to normalize and calculate ELISA units, with the standard curve as a reference.

Reverse cumulative distribution plots for each protein (Mcp, TmpH, ORF059, ORF096, ORF123) were assigned from direct OD values according to Miura et al. (25–27). Statistical significance determined with t-test for all combinations of proteins, two-tailed, adjusted for multiple comparisons with Bonferroni method.

### Murine Protein-Specific Sera

To obtain phage protein-specific sera, C57BL/6J male mice (Mossakowski Medical Research Centre, Polish Academy of Sciences, Warsaw) were administered subcutaneously three doses of highly purified proteins, 200 µg/mouse each on days 0, 20 and 40. Blood was collected from the orbital vein up to 7 days after the last dose.

Serum was collected into tubes (BD SST II Advance), left for 1 hour at RT to clot and separated from the clot by centrifugation (10 min, 2000 g) and stored at -20°C for further use. Plasma was collected into heparinized tubes and separated from blood cells by double centrifugation (10 minutes 2,250 g and 10 minutes 10,000 g) and stored at -20°C for further use.

### Human Plasma of Healthy Donors

Human IgG was evaluated in plasma of 55 healthy volunteers who had never been subjected to phage therapy or involved in phage work (age range, 18 to 40 years). Blood was collected into heparinized tubes, and plasma was separated from the blood by double centrifugation at 2,250 g and 10,000 g and used for ELISA.

### Human Plasma of Patients Undergoing Phage Therapy

All blood samples were collected between 2010 and 2013 from patients suffered from infections caused by *S. aureus* bacteria sensitive to phage A3R or 676Z (please see **Supplementary Figure S3A** for more details). They were taken 1-5 days or just before starting phage therapy and after the duration treatment (please see **Supplementary Figure S3A**). The plasma were separated from heparinized blood samples by centrifugation (10 min, 1,500 g) and stored at -70°C for further use.

### Ammonium Sulfate Antibody Precipitation

Antibodies were precipitated with ammonium sulfate (50% of saturation). Samples of serum were incubated with ammonium sulfate for 30 minutes on ice. Next, the suspension was centrifuged at 10,000 rpm for 12 min at 2°C. Supernatant was removed and the pellet was suspended in PBS.

### Protein Localization in Phage Capsids in Transmission Electron Microscopy

Immunoelectron microscopy was carried out with phage A3R or 676Z at 10^8^ pfu/ml. Fifteen microliters of 10-fold-diluted phage (20 mM Tris-HCl, 75 mM NaCl, 0.5% bovine serum albumin [BSA], 0.1% Tween 20, pH 8.0) was deposited on a nickel formvar/carbon coated grid (400-mesh; Agar Scientific). Then, the grids were blocked with 1% BSA. Ammonium sulfate precipitated antibodies from phage-protein specific murine serum samples were then added, and the grids were incubated for 1 hour. Next, the primary antibody (1:32,500) was added: 10-fold dilution. Subsequently, the grids were extensively washed in PBS, and 1:50 diluted immunoconjugate gold-labeled secondary antibody (10 nm; Sigma) was deposited on the grids. The grids were incubated for 15 minutes and then again extensively washed in PBS, followed by distilled water. Finally, the samples were stained with 2% uranyl acetate and were observed with a Zeiss EM900 transmission electron microscope.

### Phage Blocking by Specific Plasma in Human Model

Plasma obtained from healthy donors (N=55) or patients (N=13) was used. As a control, fetal bovine serum was used. Each plasma sample was applied as an inactivated or non-inactivated plasma (i.e. after or without inactivation of complement). Plasma was heat-inactivated by incubation at 56°C for 1 h. For blocking of phage activity, A3R or 676Z (1x10^7^pfu/ml to 2x10^7^pfu/ml) was mixed with plasma samples (1:1) and incubated at 37°C for 1.5 h. Phage titers in the samples were determined by the routine test dilution (RTD) method. The assay was repeated three times. An example experiment with individual statistics is presented.

### Phage Blocking by Protein-Specific Sera in Murine Model

Sera obtained from immunized mice after injecting protein (as described above) were used. As a control, serum from PBS-injected mice was used. Each serum sample was applied as an inactivated or non-inactivated serum (i.e. after or without inactivation of serum complement). Sera were heat-inactivated by incubation at 56°C for 1 h. For blocking of phage activity, A3R or 676Z (10^9^ pfu/ml) was mixed with each serum sample (1:1) and incubated at 37°C for 1.5 h. Phage titers in the samples were determined by the RTD method. Six samples per group (N = 6) were tested, and the assay was repeated three times. An example experiment with individual statistics is presented.

### Intraperitoneal Immunization of Mice and Phage-Specific Reference Plasma Samples

C57BL/6J male mice (N=6) (Medical University of Bialystok) were administered intraperitoneally (IP) three doses of highly purified phage preparations (A3R or 676Z), 10^10^ pfu per mouse each on days 0, 20 and 50. Blood samples were collected from the tail vein on days 1, 3, 5, 7, 10, 15, 20, 22, 25, 30, 35, 40, 45, 50, 52, 55, 60, 62, 65, 70, 80, 90, 100, 110, 120, 130, 140, 150 and plasma was separated as described above.

To obtain phage-specific reference plasma samples, C57BL/6J male mice (Medical University of Bialystok) were administered IP three doses (10^10^ pfu per mouse) of highly purified phage preparations, on days 0, 20 and 50. Blood samples were collected from the tail vein on days 55 and 100 and plasma was separated as described above.

### Phage – Neutralizing Potential of Antiphage A3R and 676Z Antibodies

Plasma from 55 healthy donors or patients undergoing phage therapy were examined in a neutralization test of *S. aureus* A3R and 676Z phages. The diluted plasma (1:10) were incubated with phages (10^9^ PFU/ml) at 37°C for 90 minutes. After incubation, the mixture was diluted 100-fold with cold-enriched broth. The phage titer was estimated by the double agar layer method described by Adams (24).

### Bioinformatic Analysis of Protein Structure

The structure models were generated with I-TASSER 5.1 with GNU Parallel (28–30) and visualized with PyMOL (The PyMOL Molecular Graphics System, Version 1.2r3pre, Schrödinger, LLC). The online server and the version on a local computer were used.

### Microarray Gene Expression Profiling

The cell line SC (human peripheral blood macrophages, ATCC CRL-9855) were cultured in appropriate media recommended by the manufacturer with and without protein solutions containing staphylococcal phage capsid derived proteins: Mcp (major capsid protein in phages A3R and 676Z) or TmpH (0.5 µM) and T4 phage capsid derived proteins: gp23 (major capsid protein in T4 phage) or Hoc (0.5 µM), for 6 h. This time was chosen to allow for the development of cellular responses to external factors at the cellular gene expression level, based on preliminary tests. Control cells were cultured with an equivalent amount of albumin (Sigma, Poznan, Poland). After incubation, cells were harvested and the total RNA was immediately isolated with RNeasy Mini Kit (Qiagen). For this study, we used SurePrint G3 Human Gene Expression v3 8 × 60 K Microarrays (Agilent Technologies, Santa Clara, CA, USA). The One-Color Microarray-Based Gene Expression Analysis Protocol (version 6.9.1) was used to process the arrays.

After amplification, the total RNA was labeled with Cy3 using the Low Input Quick Amp Labeling Kit (Agilent Technologies, USA). The labeled RNA was purified (RNeasy Kit, Qiagen, USA), and the RNA yield [nanograms of complementary RNA (cRNA)] as well as specific activity (picomoles of Cy3 per microgram of cRNA) were measured using a NanoQuant plate (Tecan Group, Germany) in an Infinite 200 PRO reader. Next, the labeled cRNA was fragmented and placed on the microarray slide after mixing with the hybridization buffer. Microarrays were incubated for 17 h at 65°C and then washed twice in GE wash buffer (Agilent). Agilent’s High-Resolution C Microarray Scanner was used to scan the slides according to the 8 × 60 K array format. The scanned images were analyzed with the Agilent Feature Extraction software v. 12.1 (Agilent Technologies, Santa Clara, CA, USA). The final analysis included dye normalization (linear and LOWESS), background subtractions, and filtering of outlier spots.

### Bioinformatics Analysis of Microarrays

GeneSpring GX 13 program (Agilent, USA) was used to further analyze the data after extraction. The cut-off was set to 2-fold for the determination of significant differential expression (up or down regulation). A moderated Student’s t-test was used to determine significant differences, defined as p ≤ 0.02, for the gene expression.

## Results

### Search for Phage Capsid Components Exposed to Mammalian Immune Surveillance

**Figure 1:**
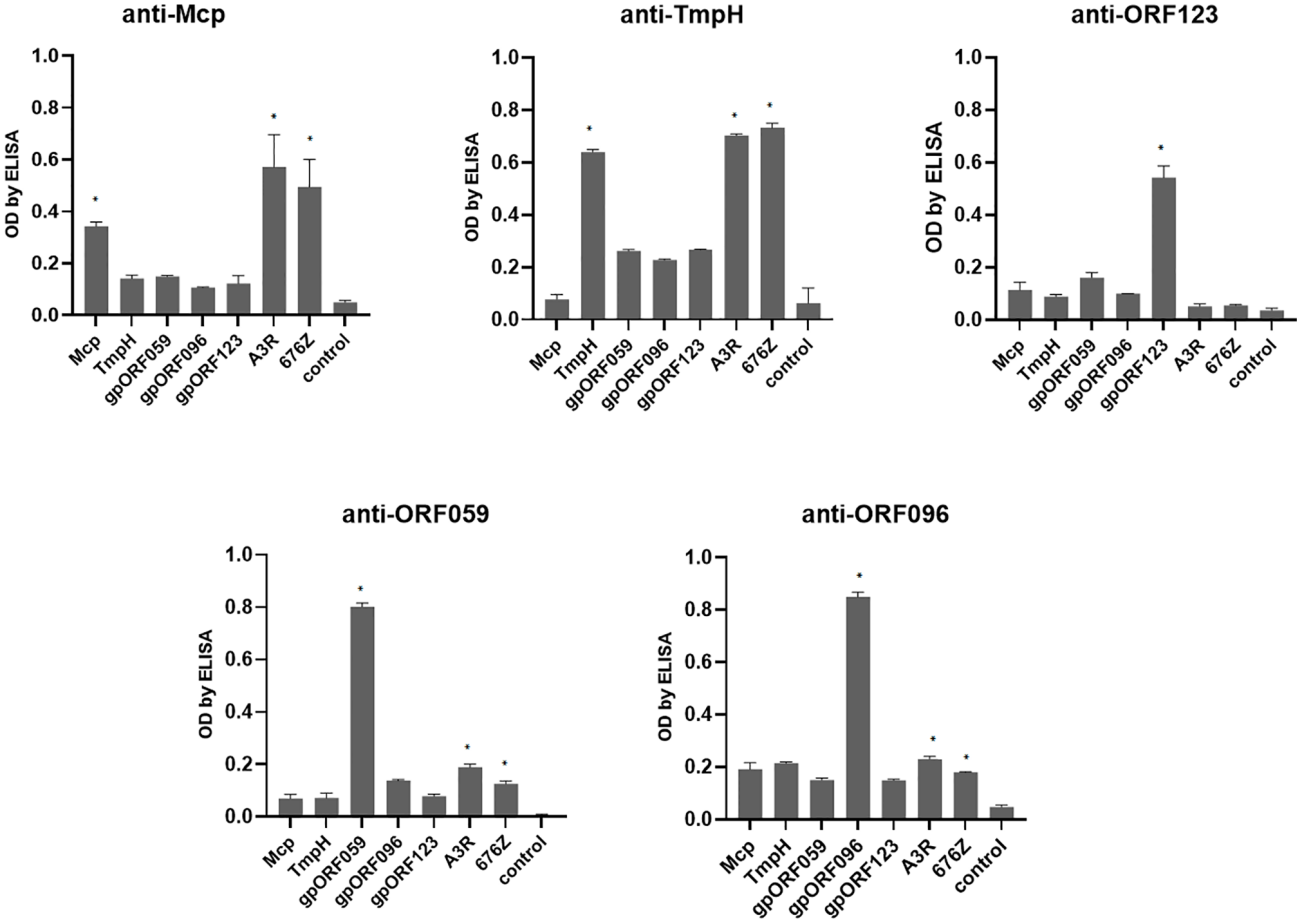
Cross-reactivity between phage protein-specific sera evaluated by enzyme-linked immunosorbent assay (ELISA). Mice were challenged subcutaneously with purified proteins: Mcp, TmpH, ORF059, ORF096 and ORF123, 200 μg per mouse; sera were collected after 40 days. As bottom antigen in ELISA purified proteins (Mcp, TmpH, OFR059, ORF096 or ORF123) or phages (A3R or 676Z) were applied. The error bars represent mean ± standard deviation (SD) from three independent experiments performed in triplets. Statistically significant differences between groups are marked with asterisks: *p < 0.005 (Kruskal-Wallis ANOVA).

Both phages, A3R and 676Z were obtained from our Therapeutic Phage Collection of the HIIET PAS and they are applied in PTU as therapeutic agents (4, 17). Therefore, we were prompted to further investigate their biological and structural features, particularly those potentially engaged in interaction with the human organism during antibacterial therapy. From the point of view of human immunity, the capsid, as the external shell of the virus, is directly exposed to immune surveillance and potentially immunogenic. We utilized annotations from the bioinformatics analysis of A3R and 676Z phage genomes (20). Key candidates were selected, starting with an obvious one (i) head major capsid protein (Mcp), (ii) tail morphogenic protein H (TmpH), then less known (iii) ORF059, (iv) ORF096 and (v) ORF123 (**Supplementary Figure S1**). We cloned all coding sequences and expressed the recombinant proteins in the *E. coli* system. These proteins were purified in 5 steps: dialysis followed by purification with a commercially available endotoxin-removal kit, EndoTrap, then high-pressure liquid chromatography (HPLC), EndoTrap and dialysis again. Purified recombinant proteins were applied subcutaneously for mouse immunization. Successful immunization yielding high-affinity antibodies was achieved (**Figure 1**). The antibody fraction was precipitated from sera after immunization with ammonium sulfate and used for further analyses.

### Localization of Structural Proteins on A3R and 676Z Phage Capsids With Immunogold TEM

**Figure 2:**
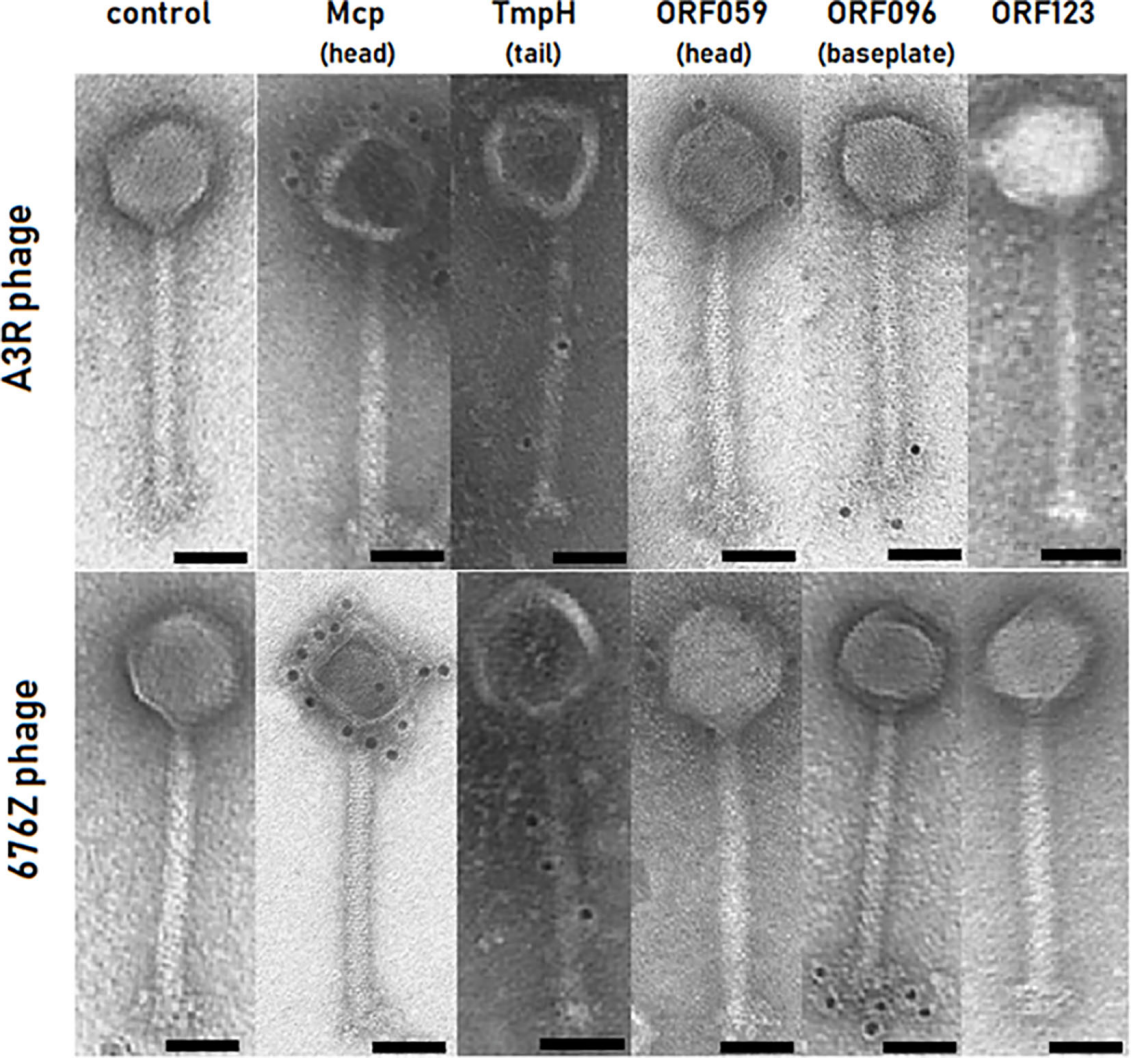
Identification of proteins’ location in A3R and 676Z phage capsids by immunogold EM technique. Primary antibodies active against each phage protein (Mcp, TmpH, ORF059, ORF096 or ORF123) were produced in mice. Secondary antibodies conjugated to 10 nm size gold nanoparticles were used. Each scale bar represents 50 nm. Exemplary TEM images.

We utilized our antibody precipitates for immunogold detection by transmission electron microscopy (TEM). Four out of five proteins were successfully detected with the immunogold TEM approach on intact phage virions. As expected, Mcp protein was detected on the head of both phages – A3R and 676Z – confirming the predicted localization (**Figure 2**). Also TmpH protein was found in the tail of both phages (**Figure 2**). For the remaining three proteins, their localization was not predicted by the annotations. Our localization study demonstrated that ORF059 is structurally exposed on the phage head (**Figure 2**) (GenBank acc. no. AFN38113.1 for A3R phage and AFN38307.1 for 676Z phage). The double checking approach (i.e. two strains of phage and two independent courses of mice immunization) was used to confirm the localization of proteins. For ORF096, the same study demonstrated its localization in the vicinity of the baseplate (**Figure 2**) (GenBank acc. no. AFN38152.1 for A3R phage and AFN38346.1 for 676Z phage). In contrast to the successfully delineated localization of these four proteins, the last one, ORF123, did not generate any immunogold signal despite multiple immunization trials (**Figure 2**). Notably, when purified protein was applied for coating the ELISA plate, the antibodies generated in mice strongly bound to ORF123, confirming specific antibody presence. Thus, we propose that ORF123 product is not exposed on the outer surface of the virion, in any of phages A3R and 676Z. The similar phenotype of both phages was shown in TEM, notably no major differences between phage A3R and 676Z were observed in micrographs (**Figure 2** panel: control).

### Antibodies Specific to Staphylococcal A3R and 676Z Phages in Healthy Human Population

**Figure 3:**
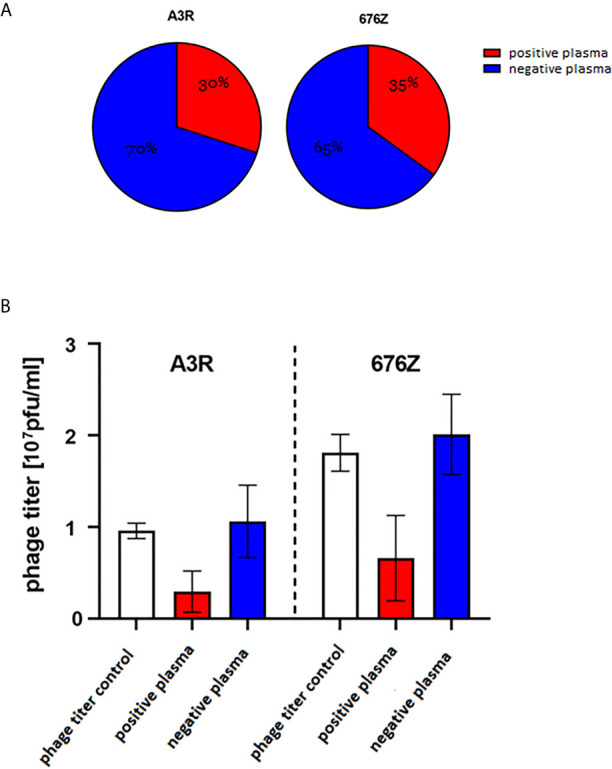
Phage-inhibitory effect of healthy humans’ plasma. Plasma were collected from 55 healthy donors never subjected to phage therapy, **(A)** frequency of phage-inhibitory effect in the investigated donors, **(B)** strength of phage inhibition by the investigated plasma: average phage titer after incubation with positive (containing phage-neutralizing antibodies) and negative (not containing phage-neutralizing antibodies) plasma. Positive plasma – inactivating the phage (phage titer after incubation was lower than the range of the negative samples); 16 samples were classified into this group in the case of A3R and 19 in 676Z. Negative plasma – not inactivating phage (phage titer after incubation was within the range: mean value of the control +/- 2 x SD); 39 samples could be classified into this group in the case of A3R and 36 in 676Z. Control was calculated from titers of the same phage preparation incubated with commercial fetal bovine serum samples (N = 8). The error bars represent mean ± standard deviation (SD).

The human population is constantly exposed to natural contact with bacteriophages that constitute a major part of the human natural virome, the phageome (31, 32) and those commonly present in food and water (33). These exposures may result in the production of phage-specific antibodies (natural antibodies) (14, 34–36). Pre-existing natural antibodies may potentially enter in interactions with a therapeutic phage (16). Therefore we determined the frequency of staphylococcal phage-specific (neutralizing) antibodies in humans, specifically in healthy volunteers who had not been subjected to phage therapy (**Figure 3**) (N=55, the same collection of plasma as previously used for a similar study by Dąbrowska et al. on T4 coliphage and Hodyra-Stefaniak et al. on anti-pseudomonas phages) (14, 36). Blood plasma were collected from healthy donors and incubated with anti-staphylococcal bacteriophages A3R or 676Z to identify neutralizing activity of phage-specific antibodies (positive plasma). Results were compared to the phage titer control and classified into two groups. Negative plasma were those not inactivating the phage (phage titer after incubation was within the range of the mean value of the control ± 2 SDs). Positive plasma were those inactivating the phage (phage titer after incubation was lower than the range of the negative samples) (14, 36). In as many as 30% (16 out of 55) of plasma samples in A3R phage and 35% (19 out of 55) of plasma samples in 676Z phage, a decrease of phage antibacterial activity in the presence of investigated plasma was observed (**Figure 3**). Average phage titer after incubation with positive plasma remained in the same order of magnitude as controls, that is: 27.65% of the initial phage titer for A3R phage and 32.78% in 676Z phage (**Figure 3**). This suggests that the general potency of pre-existing (in human population) natural antibodies to neutralize these phages is not high, and the phages may have sufficient therapeutic potential. No significant differences between phages A3R and 676Z were observed in the general comparison. Some variability was however observed among human individuals’ plasma reactivity towards A3R versus 676Z phage; 13 plasma samples out of 55 studied (23.6%) inhibited only one phage, with no effect on the other one (**Supplementary Table S1**). This demonstrates some antigenic differences between these two otherwise closely related therapeutic phages, suggesting that similar phages can be complementary (e.g. as phage cocktail components) in cases where specific antibodies hinder therapeutic application of phage.

### Structural Phage Proteins as Natural Immunogens in the Human Population

**Figure 4:**
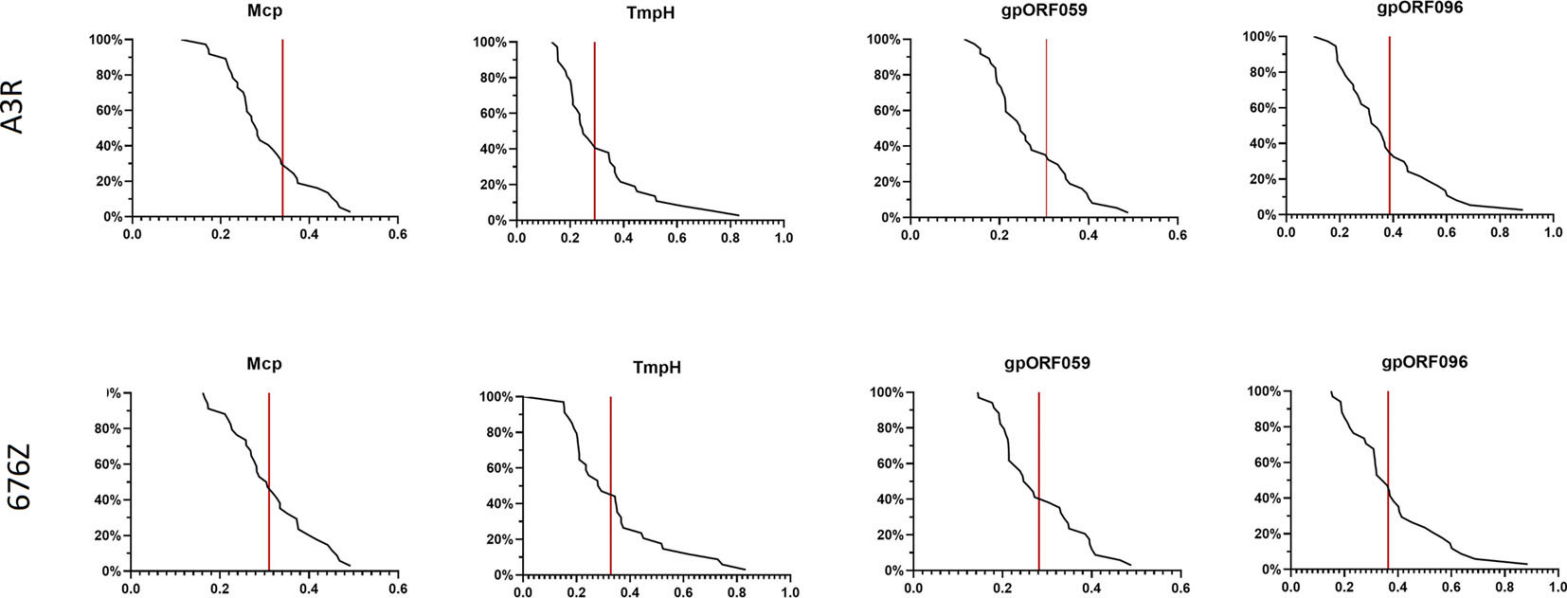
Frequency of antibodies specific to selected phage structural proteins Mcp, TmpH, ORF059, ORF096 in human population. Reverse cumulative distribution plots for capsid proteins (Mcp, TmpH, ORF059, ORF096) in cases of both phages (A3R and 676Z) were assigned to the human plasma, according to the work of Miura et al. (25). Plasma samples (N=55) were tested at 1:100 dilution in ELISA. Vertical line represents cutoff value for nonimmunized individuals. Statistical significance determined with t-test for all combinations of proteins, two-tailed, adjusted for multiple comparisons with Bonferroni method, cut-off p = 0.05.

In further studies we examined healthy human population for specific fractions of antibodies that recognize the studied structural phage proteins. The levels of antibodies specific to the four investigated proteins Mcp, TmpH, ORF059, and ORF096 (**Figure 2**) were assessed in healthy human donors’ plasma that were previously identified as containing phage neutralizing antibodies (**Figure 3**). They were compared by ELISA and reverse cumulative plotting; areas under the curve in the part representing X axis values over the control (red lines) should be compared to identify possible immunization. (**Figure 4**). No statistically significant difference was observed in levels of specific IgG between measured proteins (p > 0.05 in each combination). It indicated that no substantial discrepancy in population immunity to these phage proteins were detected (**Figure 4**). This indicates that none of the tested proteins (Mcp, TmpH, ORF059, ORF096) contributed significantly to A3R and 676Z phage immunogenicity at the population level, though specific antibodies for all studied proteins were identified in the investigated population. Nevertheless, we cannot exclude the possibility of other protein (present on the phage capsid) able to evoke immune response to the whole phage (14, 19, 37, 38).

### Phage-Related Antibodies in Patients Subjected to Staphylococcal Phage Therapy

**Figure 5:**
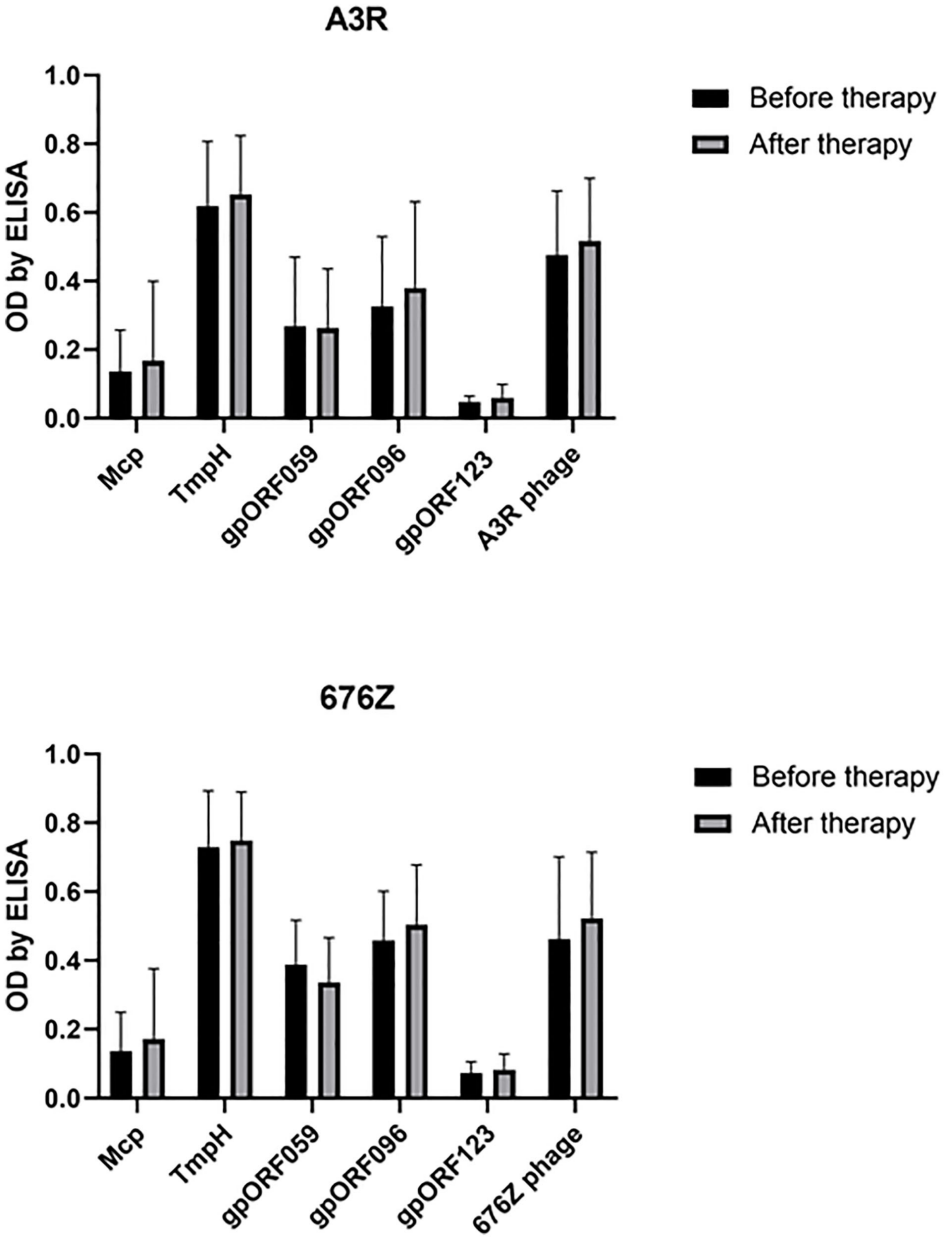
Phage protein-specific antibodies (anti-Mcp, anti-TmpH, anti-ORF059, anti-ORF096 and antibodies specific to non-structural ORF123 - as a negative control) in patients treated with A3R or 676Z phage. Plasma samples before phage therapy and after phage therapy were tested at 1:100 dilution in ELISA. The error bars represent mean ± standard deviation (SD).

Antibodies specific to selected structural proteins of A3R and 676Z phage were further investigated in patients treated at the PTU against staphylococcal infections. In general, we did not observe increased specific immune responses to A3R and 676Z phages. First, IgG antibodies specific to the structural phage proteins investigated herein (Mcp, TmpH, ORF059, ORF096) were assessed in the same plasma from patients undergoing phage therapy. No significant change in the specific IgG after phage therapy was observed for any of the investigated phage proteins (**Figure 5**). This indicates that none of the tested proteins (Mcp, TmpH, ORF059, ORF096), even if represented in high copy numbers and strongly exposed on phage capsids, was capable of inducing a significant immune response in the patients after phage therapy (17, 18).

### Immune Response of Mice Challenged With Intact Phages

**Figure 6:**
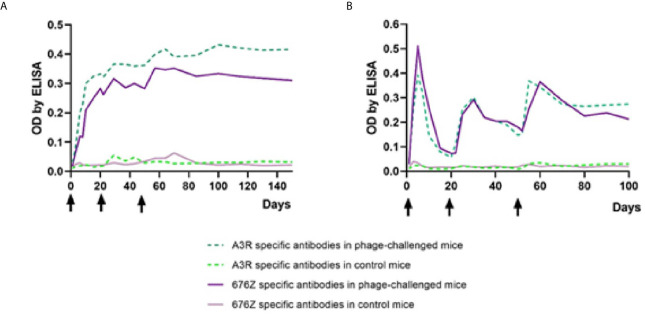
Kinetics of phage-specific antibody production in mice treated with phages A3R and 676Z. IgG **(A)** and IgM **(B)** levels were evaluated ELISA. Mice (N=6) were challenged intraperitoneally with purified preparations of phages A3R or 676Z 10^10^ pfu per mouse or injected with the same amount of PBS (control) on days 0, 20 and 50 (arrows). This experiment was repeated 2 or 3 times (IgG and IgM, respectively) with concordant results. One representative experiment is presented in the figure.

A high-dose phage challenge to evoke immunization, not applicable in humans, was therefore tested in mice. A high load of phage was applied into the peritoneal cavity of mice, intentionally forcing the immune defense. Mice were inoculated with purified phage preparations (A3R or 676Z, 10^10^ pfu per mouse) on days 0, 20 and 50. These animals were sampled for 100 days to evaluate IgM levels and for 150 days to evaluate IgG levels of specific antibodies to both phages. Kinetics of IgM and IgG increase in blood are presented in **Figure 6**. In general, challenging mice with the phages resulted in an evident increase of phage-specific antibody level in murine blood plasma. IgM level peaked as early as on day 5 for both phages. This peak was followed by a decrease of IgM to relatively low values. After secondary (day 20) and tertiary (day 50) administration of phages, IgM level peaked temporarily, and stabilized significantly above phage-specific IgM levels in control mice. Phage-specific IgG levels, in turn, increased after primary challenge with the phages without a further decrease (a marked increase was observed already in the first week, then continued within weeks 2 and 3). IgG reached a stable, high concentration approximately within the second month after the first challenge; only a small increase was achieved by the third application on day 50. The kinetics of phage-specific IgG and IgM induction were highly similar for both phages; minor differences between phages were not statistically significant. Antibodies specific to tested phages were able to neutralize phage virions (data not shown).

### Induction of Specific Antibodies in Mice by Structural Proteins of A3R and 676Z Phages

**Figure 7:**
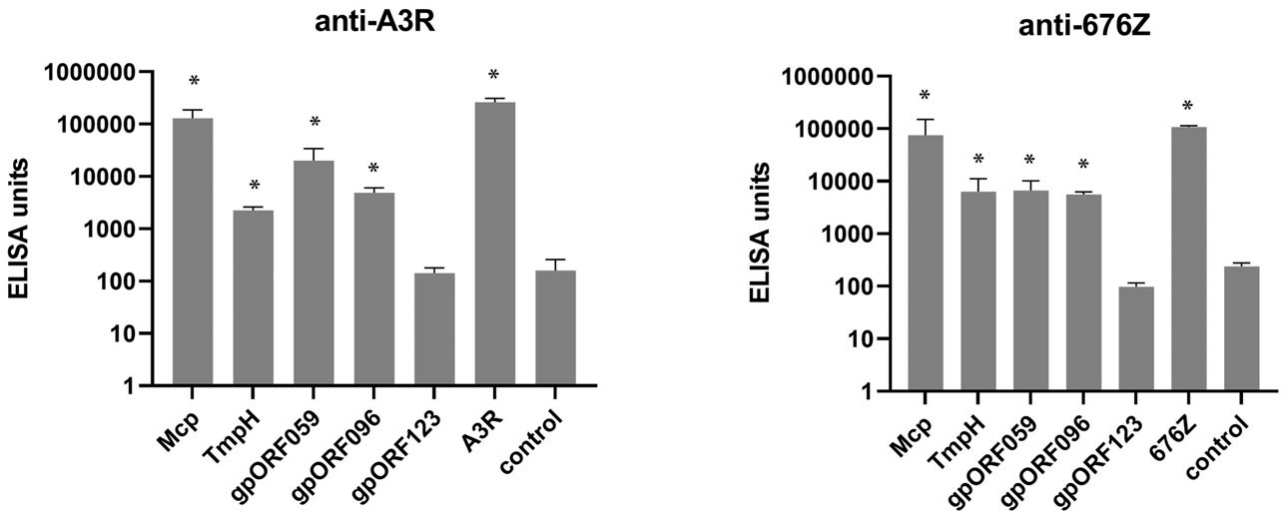
Individual immunogenicity of five selected proteins of phage A3R and 676Z in mice treated with the phage intraperitioneally assessed by IgG ELISA units. Mice (N=6) were challenged with purified preparations of phages A3R or 676Z, 10^10^ pfu per mouse. Separated plasma samples from these mice were collected after 100 days. Samples were examined for IgG antibodies specific to selected structural proteins: Mcp, TmpH, gpORF059 and gpORF096. ORF123 was tested as a negative control (non-structural protein). Results were normalized and ELISA units were calculated for each sample according to Miura et al. (25, 26). The error bars represent mean ± standard deviation (SD). Statistically significant differences between groups and control are marked with asterisks: *p < 0.05, Kruskal-Wallis ANOVA.

Next, we tested the immunogenicity of four proteins exposed as structural elements on the phage virions and one (ORF123) was added as a negative control (not detected as structural element in previous studies: **Figure 2**), as we described above. Protein-specific IgG was tested in murine plasma collected on day 100 of the experiment described in the previous section (**Figure 6**). IgG levels were compared by standardized ELISA units (14, 15, 25, 26) with isolated recombinant phage proteins or phage as bottom antigens (**Figure 7**). As expected, Mcp protein, which is present on phage heads in many copies (major structural protein), induced the highest levels of specific antibodies (**Figure 7**). Structural protein ORF096, located on the baseplate, also significantly contributed to the immunogenic effect in A3R phage. The other structural proteins TmpH and ORF059 induced protein-specific IgG production, although with some variability between phages and individual animals (**Figure 7**). We demonstrated for the first time that two of the newly localized capsid structures are capable of inducing an immune response not only in humans, but also in mice.

### Effect of Antibodies Specific to Phage Proteins Mcp, TmpH, ORF059, ORF096 on Antimicrobial Activity of Phage A3R and 676Z

**Figure 8:**
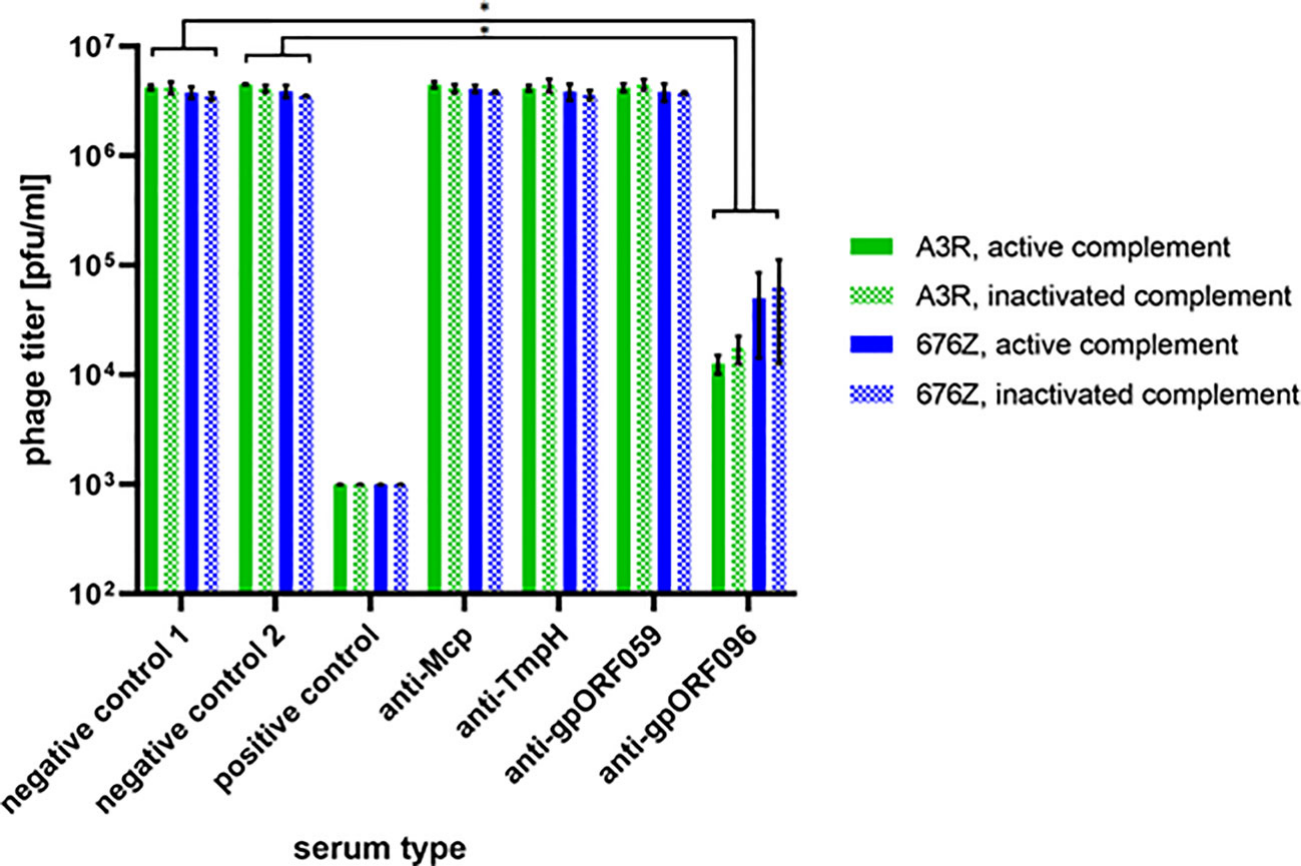
The effect of phage protein-specific sera on antibacterial activity of A3R and 676Z phages. Phages were incubated with non-inactivated (active complement) or heat-inactivated (inactive complement) murine sera specific to proteins Mcp, TmpH, ORF059, and ORF096. As a negative control we used commercial fetal bovine serum (negative control 1) or murine serum specific to the non-structural phage protein ORF123 (negative control 2). The positive control was plasma containing phage-specific antibodies induced by whole phages A3R or 676Z (day 100 after challenge). The error bars represent mean ± standard deviation (SD) from three independent experiments performed in triplets. Statistically significant differences between groups are marked with asterisks: *p ≤ 0.0005, two-tailed Welch’s t-test.

The potential effect of antibodies specific to individual structural phage proteins on antibacterial activity of whole phages was investigated using protein-specific sera developed after challenging mice with recombinant, isolated and highly purified phage proteins. Phages were incubated with protein-specific sera *ex vivo*. In the case of Mcp, TmpH, ORF059 and nonstructural ORF123 (negative control 2) protein-specific sera did not inhibit activity of bacteriophages A3R or 676Z. However, serum specific to ORF096 strongly inhibited phage ability to lyse bacteria, as revealed by the significant decrease of active phage titer (remaining titer was 0.4% or 1.8% of the initial titer in phage A3R and 676Z, respectively) (**Figure 8**). This demonstrates significant differences in the effect that protein-specific antibodies exert on phage activity and suggests that ORF096, of so far unknown precise location, might be exposed for immune recognition. It further suggests that ORF096 is involved in the process of infection of bacterial cells by bacteriophages A3R and 676Z.

## Discussion

Induction of specific antibodies by bacteriophages has been observed in many previous studies (9, 13–15, 22). However, reports presenting detailed kinetics of antibody production, frequency of antibodies specific to particular phages in the human population, or individual immunogenicity of phage proteins are very rare. Natural (not related to therapeutic use of phage) antibodies specific to particular phage have been observed in humans but their frequencies in the population may vary. Kucharewicz-Krukowska and Slopek (16) reported the frequency of natural antibodies specific to some therapeutic phages as approximately 23%, while the frequency of T4-phage neutralizing antibodies in humans was estimated at 81% (14). In our previous study with PB1-like phages, 11%, 15%, and 40% of the same collection of human plasma were able to inhibit LMA2, F8, and DP1 phage, respectively (36).

Of note, the importance of phage-specific antibodies goes beyond potential neutralization of phage. Specific elements of the immunity cooperate with the non-specific ones, and this cooperation may strongly affect phage bioavailability (39). Phages are commonly filtered by elements of the mononuclear phagocyte system, even when no specific response to phage has been developed. Importantly, when specific antibodies are present in the system, they bind (opsonize) phage virions making them more `visible` to the innate immunity. This phenomenon represents the common type of phagocytosis: Fc-receptor mediated phagocytosis, where Fc regions of antigen-specific antibodies enhance engulfment of the antigen. Immune cell interactions with bacteriophages are possible at distant sites such as e.g. splenocytes and Kupffer cells (40, 41). Antibodies that opsonize bacteriophages facilitate phagocytosis and may indirectly contribute to phage neutralization *in vivo*. Also, complement system as the innate immunity part, may cooperate with antibodies that may trigger classic complement pathway against an antigen recognized by a specific antibody. This is again a common immunological pathway that contributes to removal of foreign objects from the system (42), which engage specific antibodies cooperating with innate immunity into phage neutralization (41). Further, phage preparations may potentially contain remains of endotoxins, and thus induce some cytokine production (8), which may further increase specific immune response *via* innate part of the immune system.

Here we investigated two closely-related therapeutic bacteriophages active against *Staphylococcus aureus* (A3R and 676Z), and we observed pre-existing (natural) antibodies able to neutralize these phages in humans: A3R (30%) and 676Z (35%). These levels of phage neutralization resulted from many unspecified (polyclonal) antibodies contained in the samples. This means that a summary effect of either neutralizing or ‘partially neutralizing’ or non-neutralizing antibodies was observed. Importantly, inhibition of phage activity was *not* markedly pronounced; phage titer after incubation with positive plasma remained in almost the same order of magnitude as controls (it achieved on average 28% of initial phage titer in A3R and 33% in 676Z) (**Figure 3**). Although A3R phage and 676Z phage are closely related, reactivity of specific individuals’ plasma with A3R and 676Z differed in as much as 23.6% of investigated individuals (meaning: one phage was inhibited with no effect on the other one, **Supplementary Table S1**), which demonstrates antigenic differences between these two otherwise similar therapeutic phages. This further suggests that in the case of a high level of a neutralizing specific response to one phage, another phage could be used as a replacement in the therapy.

Searching for the origin of natural antibodies in naïve humans, we have identified two proteins of so far unknown precise location in staphylococcal phage, as immunity inducer candidates. We raised in a murine model antibodies against them and provided precise (immunogold EM) localization of these 2 candidate proteins, one of them in the head (ORF059) and the other one in the baseplate (ORF096), thus both in a position on the virion surface exposed to immune surveillance. We further demonstrated that these proteins contributed to human natural immunization, together with two other tested components of staphylococcal phage, major capsid protein Mcp and tail protein TmpH. In addition, these four phage proteins can be considered as targets for possible surface modifications in therapeutic phages A3R and 676Z to engineer phage properties in the future.

Importantly, differences in the strength of protein specific sera reactions with phages as bottom antigens were observed (by ELISA) (**Figure 1**). These may result either from possible problems with some protein folding, or from differences in antigen amounts on phage virions. Major capsid and tail proteins are high-copy elements of phage virions, while proteins ORF059 and ORF096 are rather low copy elements. Here we observed that sera specific to the low-copy proteins reacted weaker when whole phages were used as bottom antigens. Sera specific to high-copy proteins (Mcp, TmpH) reacted intensively when whole phages were used as bottom antigens. However at this stage of the study, it is not possible to determine which effect contributed in what extend to the final result. Possible problems with conformation of recombinant proteins used in this study should be considered as the study limitation.

Importantly, Mcp and TmpH as the highly immunogenic and high-copy proteins were tested for their possible effects on immunological cells at the gene expression level (DNA microarray assay). This revealed no significant changes in gene expression after exposition of human peripheral blood macrophages (SC) to the proteins, indicating that proteins are neutral for the cells. Accordingly, reference proteins of a model phage T4 that had previously been demonstrated as highly immunogenic, had no detectable effect on gene expression profiles in human cells (cell line). This contributes to safety assessments of therapeutic phages demonstrating that major elements of phage capsids had no negative effects on the human cells (**Supplementary Table S2**). This study included a single cell line (SC) which is a limitation due to the fact that other types of cells may display different patterns of response to phage proteins. Notably, selected phage proteins were tested only, while other phage proteins may exert physiological effects not yet specified.

We included mice into antibody production analysis because high-dose antigenic stimulation and specific protein stimulation could not be achieved in humans. The doses applied to mice were high enough to achieve effective immunization. First, we compared the overall ability of whole phages to induce specific IgM and IgG. We observed that both phages induced in mice a typical, rapid increase of IgM with a maximum peak around day 5, later declining, and a subsequent increase of IgG, which achieved the highest concentrations within the third week, and then stabilized (**Figure 6**). Thus, bacteriophages efficiently induced production of specific IgM and IgG antibodies in mice, and the kinetics of these antibodies were typical, as observed for many other protein antigens and in other therapeutic phage (F8) (36).

For phage therapy purposes, pharmacokinetic scaling of bacteriophage dose from animals to humans would be useful, including differences in administration routes. To estimate adequate phage dose in humans, we propose the simplification of volumes as proportional to weight of species (15). In our previous studies of oral administration route in mouse models, we calculated that 2 × 10^10^ pfu per mouse daily, equals 7 × 10^13^ pfu per human patient daily (15). Also in this study we evoked IgG and IgM in mice with the phage doses 10^10^ pfu per mouse intraperitioneally each (three doses), which would equal 3.5 × 10^13^ per a human patient. Intraperitoneal route of phage administration is not applied in human treatment. The amount of phages typically administered (orally or topically) in Phage Therapy Unit (Wrocław, Poland), is significantly lower and ranges between 3 × 10^7^ to 6 × 10^10^ pfu per patient daily (4). In our study we found this amount too low to induce significant increase in the phage-specific antibodies (**Figure 5** and **Supplementary Figure S3**).

Next, we compared the immune response elicited by phage structural proteins after administration of whole phage particles to mice. Similar to that in T4 phage (14), major structural protein was the major element of the capsid that induced specific antibodies (**Figure 7**). This advocates that proteins present in phage virions in many copies and strongly exposed to external interactions (such as major capsid proteins, Mcp) are the most efficient inducers of specific antibody responses to phage. On the other hand, these Mcp-specific antibodies may have no effect on phage antibacterial activity (**Figure 8**). Also, other structural phage proteins located on the phage head or tail surface did not affect phage ability to kill bacteria. However, phage-neutralizing activity was observed in antibodies specific to gpORF096 that was located in the baseplate (**Figure 8**). The ability of phage neutralization by anti-gpORF096 antibodies is probably related to the function of this protein and together with its location it suggests that gpORF096 is engaged in the process of infection, possibly as a ligand for bacterial receptors responsible for phage adsorption on the bacterial surface. This will be further investigated in our future studies.

Although both tested phages, A3R and 676Z, in which we identified novel structural immunogens are closely related, other *Hellereviridae* of the human phageome could contribute to measured antibody levels, by means of cross-reactivity. Independent of the origin of naturally occurring immunity against staphylococcal phage, in the experiences of physicians its impact on therapeutic efficacy appeared negligible, and did not preclude favorable outcome of phage therapy (17, 18). In further studies individual analysis of IgG antibodies specific to the proteins investigated herein separately (Mcp, TmpH, ORF059, ORF096) in patients plasma samples revealed no significant increase after phage therapy (**Figure 5** and **Supplementary Figure S3**). This indicates that none of the tested proteins (Mcp, TmpH, gpORF059, gpORF096), even if represented in high copy numbers and strongly exposed on phage capsids, was capable of inducing an important immune response in the patients subjected to phage therapy. Thus, both the biological test of lytic effects of phage and the *in vitro* immunological testing together confirmed natural occurrence of anti-phage antibodies but neglected their clinical significance.

Though in the mouse model differences in individual immunogenicity of phage proteins were observed, in the population of healthy humans no significant differences were noted (**Figure 4**). Moreover, in murine model, the inhibitory activity was exerted by anti-ORF096 specific antibodies, in humans we did not observe similar effect of studied proteins. These observations may seem unexpected, but it may be related to substantial differences between the exposure of laboratory animals to investigated phages and the exposure of human individuals to phages as elements of natural microbiomes and environments. Probably the major differences are (i) routes of administration, with intraperitoneal injection in animals versus natural transcytosis in healthy humans (43), (ii) doses that can be much higher in the laboratory models (15), and (iii) the use of defined phages in the animal model, while in humans phage-specific antibodies result from multitude exposures to unspecified, possibly antigenically similar phages or even from cross-reactions to other antigens including non-phage ones. Eventually, our study demonstrated that none of these four proteins plays a particular role in the induction of antibodies at the population level; thus none potentially affects in a particular way the effectiveness of A3R and 676Z in humans. This includes gpORF096-specific antibodies, demonstrated in the animal model as phage-inhibitory. Our observation is in line with other results concerning phage-blocking activity in samples from phage therapy patients, where phage-specific antibodies were present but they did not affect phage therapy outcomes (17, 18). This suggests that one may find a ‘window’ between the specific immune response that can be elicited by high doses of parenterally applied phages (as in an animal model) and the response observed in patients treated with bacteriophages.

## Conclusions

Phages can induce phage-specific antibodies, including those neutralizing ones. However, in our experience their impact on therapeutic efficacy appeared negligible, and did not preclude favorable outcome of phage therapy. The difference between specific immune response that can be elicited by high doses of parenterally applied phages (as in an animal model) and the response observed in patients treated with bacteriophages allow for a ‘therapeutic window’.

## Data Availability Statement

The datasets presented in this study can be found in online repositories. The names of the repository/repositories and accession number(s) can be found in the article/**Supplementary Material**.

## Ethics Statement

The studies involving human participants were reviewed and approved by Bioethics Committee at the Wroclaw Medical University, Poland. The patients/participants provided their written informed consent to participate in this study. The animal study was reviewed and approved by 1st Local Committee for Experiments with the Use of Laboratory Animals, Wroclaw, Poland.

## Author Contributions

ZK performed major part of the experimental work including gene cloning, protein expression and purification, animal experiments, immunological assays, participated in data analysis and wrote the manuscript. JM, PM, MH, DL, and WK participated in experimental work including gene cloning, protein expression and purification, animal experiments, immunological assays. BO and JC purified phages and proteins. SN and MM-K performed TEM immunolocalization. PK participated in bioinformatic analysis. AG and RM covered all aspects of phage therapy, including patients’ interaction and supervision, treatment and related information analysis. AG reviewed the manuscript. MD participated in preparation of the manuscript. KD conceived and planned the study, participated in gene cloning, animal experiments, immunological assays, analysed and concluded data and reviewed the manuscript. All authors contributed to the article and approved the submitted version.

## Funding

This work was supported by the National Science Centre in Poland, grants UMO-2012/05/E/NZ6/03314, UMO-2015/19/N/NZ4/03609.

## Conflict of Interest

The authors declare that the research was conducted in the absence of any commercial or financial relationships that could be construed as a potential conflict of interest.
