# Supplementary material for: Immune Response to Therapeutic Staphylococcal Bacteriophages in Mammals: Kinetics of Induction, Immunogenic Structural Proteins, Natural and Induced Antibodies
Source: Front Immunol. 2021 Jun 14;12:639570. doi: 10.3389/fimmu.2021.639570 (PMC8236893; doi:10.3389/fimmu.2021.639570)
Supplement: Supplementary file 1 [file DataSheet_1.docx]

***Supplementary information***

**Predicted protein models revealed by bioinformatic analysis**

By the immunogold EM technique, we confirmed the location of Mcp and TmpH proteins in staphylococcal phages A3R and 676Z and we identified functions of ORF059 as the head protein and ORF096 as the baseplate-region protein. Because their structures are unknown, we performed protein modeling analysis. Here we present the results of the predicted models for proteins Mcp, TmpH, ORF059, ORF096 and ORF123 not detected as a structural one (Fig. S1).

**
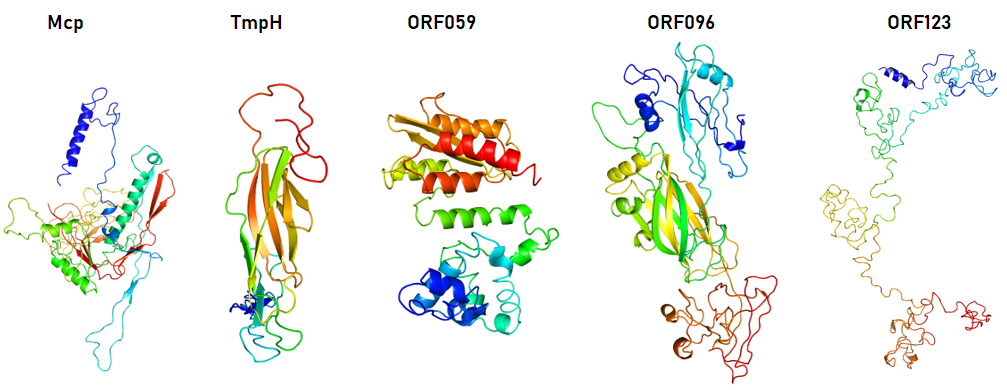
**

Supplementary Figure S1. Predicted structures of Mcp, TmpH, ORF059, ORF096 proteins present on phage capsids and ORF123 non detected on phage capsids. C-score for Mcp model was 0.93, TmpH -2.95, ORF059 -4.63, ORF096 -2.62, ORF123 -1.61, range of [-5, 2]. All structures were derived from PDB files. Analysis was performed by SWISS-MODEL software and visualized in PyMOL.

**Phage chromatography**

Gel filtration on Sepharose 4B was applied to purified phage preparations for immunological tests.


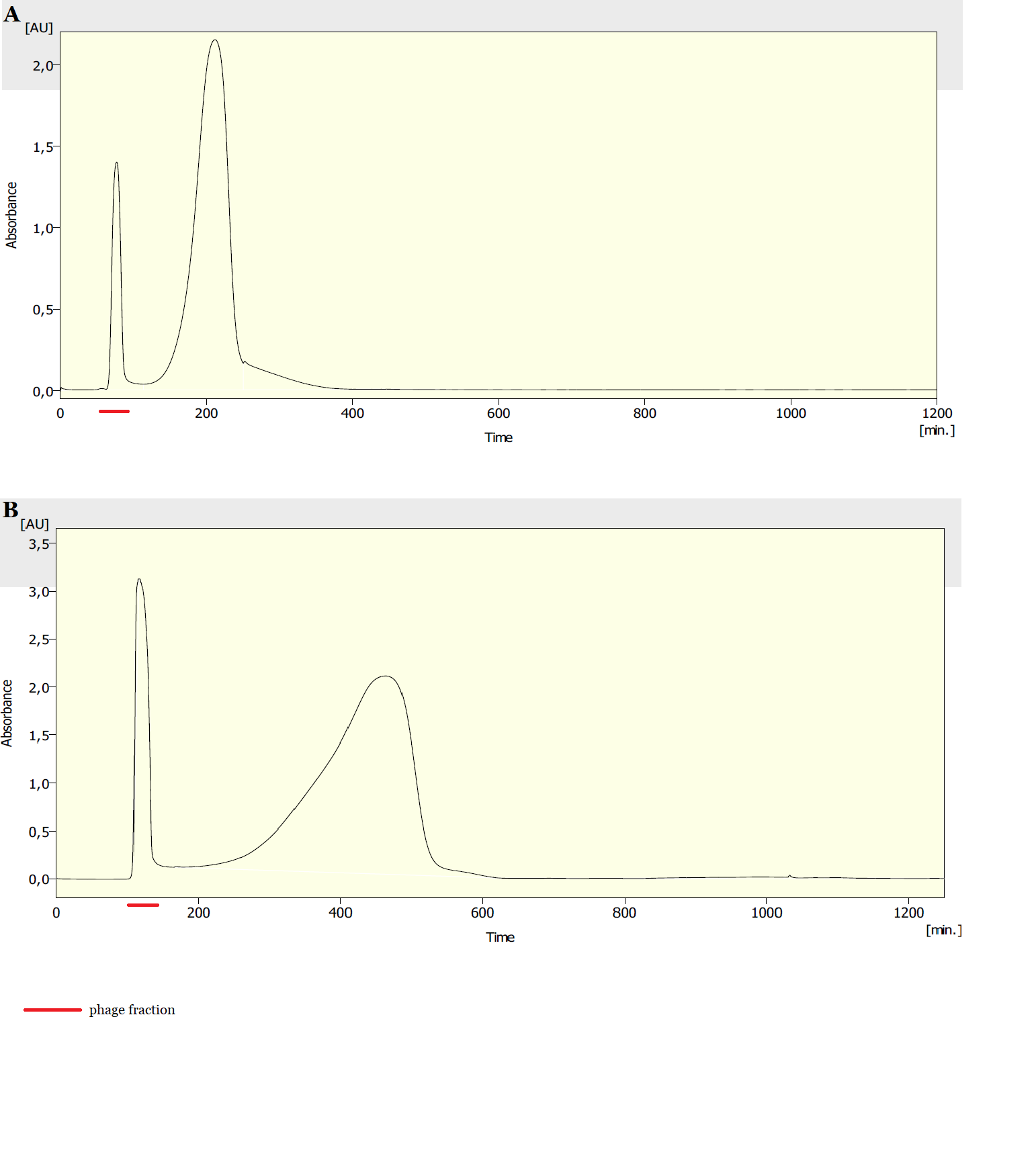


Supplementary Figure S2. Chromatograms for phages A3R (A) and 676Z (B). Size exclusion chromatography on Sepharose 4B resin (Sigma).

**Phage – neutralizing potential of antiphage A3R and 676Z antibodies**

Analysis of the compatibility between the response of plasma of 55 healthy donors with phage A3R and 676Z was performed. Plasma of 55 healthy donors (volunteers) were used for the experiments. The plasma were incubated with bacteriophage A3R or 676Z. Negative plasma (marked with minus sign) were those not inactivating the phage (phage titer after incubation was within the range of the mean value of the control ± 2 SDs). Positive plasma (marked with plus sign) were those inactivating the phage (phage titer after incubation was lower than the range of the negative samples) (14, 36). Comparison of the reactivity of the plasma with the two tested phages revealed that in 13 cases out of 55 subjects (23.6%) there is a difference between the sensitivity of A3R and 676Z phages to neutralizing plasma activity (Table S1). This indicates the differences between these two phages and also their potential complementation as active antibacterial agents used *in vivo.*

|  | **Phage** |  | **Compatibility** |
| --- | --- | --- | --- |
| plasma no. | A3R | 676Z |  |
| 1 | - | - | Y |
| 2 | - | + | N |
| 3 | + | + | Y |
| 4 | - | - | Y |
| 5 | + | + | Y |
| 6 | - | - | Y |
| 7 | - | - | Y |
| 8 | - | - | Y |
| 9 | + | + | Y |
| 10 | - | - | Y |
| 11 | - | - | Y |
| 12 | + | - | N |
| 13 | - | - | Y |
| 14 | - | - | Y |
| 15 | + | + | Y |
| 16 | - | - | Y |
| 17 | - | - | Y |
| 18 | + | + | Y |
| 19 | + | - | N |
| 20 | - | - | Y |
| 21 | + | - | N |
| 22 | - | + | N |
| 23 | + | + | Y |
| 24 | + | + | Y |
| 25 | + | - | N |
| 26 | - | - | Y |
| 27 | + | + | Y |
| 28 | + | - | N |
| 29 | - | - | Y |
| 30 | - | - | Y |
| 31 | - | - | Y |
| 32 | - | - | Y |
| 33 | - | - | Y |
| 34 | - | - | Y |
| 35 | + | + | Y |
| 36 | - | - | Y |
| 37 | - | - | Y |
| 38 | - | + | N |
| 39 | + | + | Y |
| 40 | - | - | Y |
| 41 | - | - | Y |
| 42 | + | + | Y |
| 43 | - | - | Y |
| 44 | - | + | N |
| 45 | - | - | Y |
| 46 | - | - | Y |
| 47 | - | + | N |
| 48 | - | - | Y |
| 49 | - | - | Y |
| 50 | - | - | Y |
| 51 | - | + | N |
| 52 | - | - | Y |
| 53 | - | - | Y |
| 54 | - | + | N |
| 55 | - | + | N |

Supplementary Table S1. Analysis of the compatibility between the response of plasma of 55 healthy donors with phage A3R and 676Z was performed. Plus (+) means plasma containing blocking antibodies, minus (-) means plasma non containing blocking antibodies. Y – compliance, N – lack of compliance.

**Patients**

Adult patients with various infections (e.g., leg ulcers, chronic laryngitis) resistant to antibiotic treatment received phage treatment (PT) under the therapeutic protocol entitled “Experimental phage therapy of drug-resistant bacterial infections, including MRSA infections” (4). Patients (N = 13) treated at the Phage Therapy Unit in Wrocław, Poland with the A3R or 676Z staphylococcal phage were examined. All patients received treatment over the years 2010-2013. Specific data regarding patients examined in this study are summarized in Figure S3.


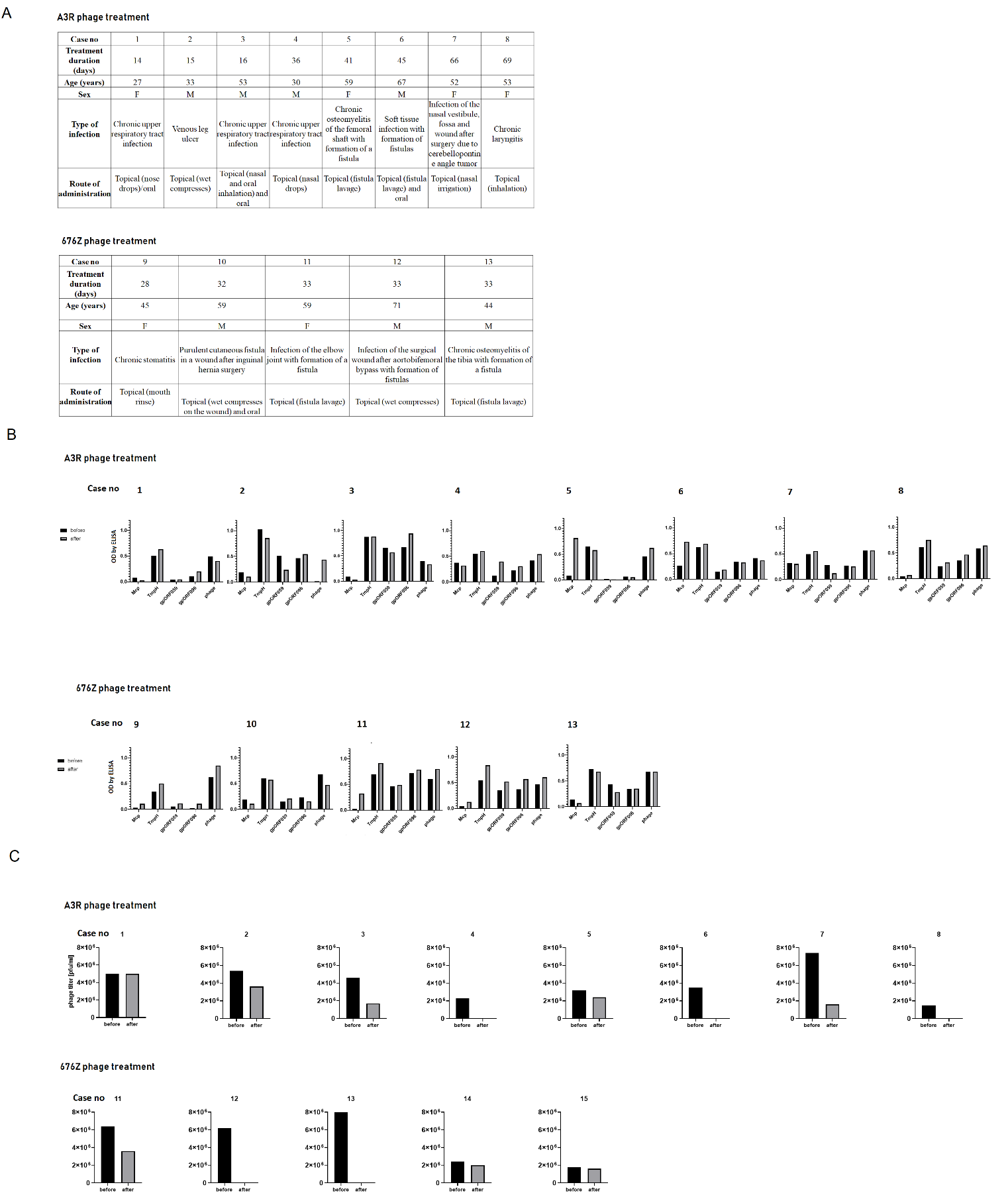


Supplementary Figure S3. A – characteristics of patients undergoing phage therapy. A3R phage treatment – cases treated by A3R phage (case no 1 – 8), 676Z phage treatment – cases treated by 676Z phage (cases no 9 – 13). B – phage protein-specific antibodies (anti-Mcp, anti-TmpH, anti-ORF059, anti-ORF096, anti-ORF123) in patients treated with A3R or 676Z phage. Plasma samples before phage therapy (“before”) and after phage therapy (“after”) were tested at 1:100 dilution in ELISA. C – phage neutralization by plasma samples of patients undergoing phage therapy. Plasma samples before phage therapy (“before”) and after phage therapy (“after”) were tested.

**Microarray gene expression profiling in human cells exposed to phage proteins**

Since investigated phage proteins were found capable for inducing immune reaction, direct response at the cellular level was also tested. For this purpose, an integrative analysis of gene expression profiles in human blood leukocytes (SC cell line) exposed to the investigated phage capsid proteins was conducted. This method provides a sensitive tool for the detection of effects that potentially active agents exert on the cells. Possible changes in immunological or other pathways can be revealed by changes in gene expression. The study included selected staphylococcal phage capsid proteins (Mcp or TmpH) and two capsid proteins of a model phage T4 (gp23 or Hoc); T4 and its proteins had previously been proposed as capable for interaction with eukaryotic cells and for modulation of immune system reactions (16, 22, 40, 41) thus gp23 and Hoc served as a reference. The cells were incubated with the investigated phage proteins and albumin (BSA) as the negative control , for 6 h. DNA microarray assay revealed no statistically significant differences in gene expression in any case: all comparisons to albumin as well as those of Mcp vs gp23, Mcp vs Hoc, TmpH vs Hoc, TmpH vs gp23 were found insignificant. Specifically, differences in gene expression were not found using the Benjamini Hochberg method for the false discovery rate (FDR) (Table S2). These results demonstrate relatively weak effects of the proteins on the human cells, even though the proteins were found immunogenic. This is in line with the safety requirements for therapeutic phages: two proteins Mcp and TmpH that build the majority of viral particles had no negative effects on the human cells, neither they exerted any other detectable effect (Table S2).

Table S2. Statistical data from the analysis of gene expression in SC cells exposed to MCP or TmpH compared to albumin (Alb) or T4 phage derived proteins (gp23 or Hoc), Benjamini – Hochberg method. Change in the expression level of genes was marked as FC; fold change. Significance of the difference in gene expression: p <0.02; p <0.001; p <0.005; p <0.001.

| **MCP vs alb** | | | | | |
| --- | --- | --- | --- | --- | --- |
|  | P all | P < 0,02 | P < 0,01 | P < 0,005 | P < 0,001 |
| FC all | 58341 | 0 | 0 | 0 | 0 |
| FC > 1.1 | 40094 | 0 | 0 | 0 | 0 |
| FC > 1.5 | 6533 | 0 | 0 | 0 | 0 |
| FC > 2.0 | 1808 | 0 | 0 | 0 | 0 |
| FC > 3.0 | 402 | 0 | 0 | 0 | 0 |
| Expected |  | 0 | 0 | 0 | 0 |
| **MCP vs gp23** | | | | | |
|  | P all | P < 0,02 | P < 0,01 | P < 0,005 | P < 0,001 |
| FC all | 58341 | 0 | 0 | 0 | 0 |
| FC > 1.1 | 32957 | 0 | 0 | 0 | 0 |
| FC > 1.5 | 5118 | 0 | 0 | 0 | 0 |
| FC > 2.0 | 1553 | 0 | 0 | 0 | 0 |
| FC > 3.0 | 291 | 0 | 0 | 0 | 0 |
| Expected |  | 0 | 0 | 0 | 0 |
| **MCP vs Hoc** | | | | | |
|  | P all | P < 0,02 | P < 0,01 | P < 0,005 | P < 0,001 |
| FC all | 58341 | 0 | 0 | 0 | 0 |
| FC > 1.1 | 43078 | 0 | 0 | 0 | 0 |
| FC > 1.5 | 22965 | 0 | 0 | 0 | 0 |
| FC > 2.0 | 6401 | 0 | 0 | 0 | 0 |
| FC > 3.0 | 1660 | 0 | 0 | 0 | 0 |
| Expected |  | 0 | 0 | 0 | 0 |
| **TmpH vs alb** | | | | | |
|  | P all | P < 0,02 | P < 0,01 | P < 0,005 | P < 0,001 |
| FC all | 58339 | 0 | 0 | 0 | 0 |
| FC > 1.1 | 36125 | 0 | 0 | 0 | 0 |
| FC > 1.5 | 5508 | 0 | 0 | 0 | 0 |
| FC > 2.0 | 1758 | 0 | 0 | 0 | 0 |
| FC > 3.0 | 410 | 0 | 0 | 0 | 0 |
| Expected |  | 0 | 0 | 0 | 0 |
| **TmpH vs Hoc** | | | | | |
|  | P all | P < 0,02 | P < 0,01 | P < 0,005 | P < 0,001 |
| FC all | 58339 | 0 | 0 | 0 | 0 |
| FC > 1.1 | 46025 | 0 | 0 | 0 | 0 |
| FC > 1.5 | 11588 | 0 | 0 | 0 | 0 |
| FC > 2.0 | 4239 | 0 | 0 | 0 | 0 |
| FC > 3.0 | 1015 | 0 | 0 | 0 | 0 |
| Expected |  | 0 | 0 | 0 | 0 |
| **TmpH vs gp23** | | | | | |
|  | P all | P < 0,02 | P < 0,01 | P < 0,005 | P < 0,001 |
| FC all | 58339 | 0 | 0 | 0 | 0 |
| FC > 1.1 | 41978 | 0 | 0 | 0 | 0 |
| FC > 1.5 | 7801 | 0 | 0 | 0 | 0 |
| FC > 2.0 | 2629 | 0 | 0 | 0 | 0 |
| FC > 3.0 | 671 | 0 | 0 | 0 | 0 |
| Expected |  | 0 | 0 | 0 | 0 |
| **gp23 vs alb** | | | | | |
|  | P all | P < 0,02 | P < 0,01 | P < 0,005 | P < 0,001 |
| FC all | 58341 | 0 | 0 | 0 | 0 |
| FC > 1.1 | 32632 | 0 | 0 | 0 | 0 |
| FC > 1.5 | 6515 | 0 | 0 | 0 | 0 |
| FC > 2.0 | 2042 | 0 | 0 | 0 | 0 |
| FC > 3.0 | 457 | 0 | 0 | 0 | 0 |
| Expected |  | 0 | 0 | 0 | 0 |
| **Hoc vs alb** | | | | | |
|  | P all | P < 0,02 | P < 0,01 | P < 0,005 | P < 0,001 |
| FC all | 58341 | 0 | 0 | 0 | 0 |
| FC > 1.1 | 45784 | 0 | 0 | 0 | 0 |
| FC > 1.5 | 14610 | 0 | 0 | 0 | 0 |
| FC > 2.0 | 4925 | 0 | 0 | 0 | 0 |
| FC > 3.0 | 1283 | 0 | 0 | 0 | 0 |
| Expected |  | 0 | 0 | 0 | 0 |
